# Value of the Sentinel Node Procedure in Pediatric Extremity Rhabdomyosarcoma: A Systematic Review and Retrospective Cohort Study

**DOI:** 10.1245/s10434-021-10035-9

**Published:** 2021-05-31

**Authors:** Bernadette Jeremiasse, Alida F. W. van der Steeg, Marta Fiocco, Monique G. G. Hobbelink, Johannes H. M. Merks, Jan Godzinski, Barry L. Shulkin, Marc H. W. A. Wijnen, Cecilia E. J. Terwisscha van Scheltinga

**Affiliations:** 1 Pediatric Surgery, Pediatric Solid Tumor Unit, Princess Maxima Center for Pediatric Oncology, Utrecht, The Netherlands; 2 Trial and Data Center, Princess Maxima Center for Pediatric Oncology, Utrecht, The Netherlands; 3 Institute of Mathematics, Leiden University, Leiden, The Netherlands; 4 Department of Biomedical Data Science, Section Medical Statistics, Leiden University Medical Center, Leiden, The Netherlands; 5 Department of Radiology and Nuclear Medicine, University Medical Center Utrecht, Utrecht, The Netherlands; 6 Pediatric Oncology, Pediatric Solid Tumor Unit, Princess Maxima Center for Pediatric Oncology, Utrecht, The Netherlands; 7 Department of Pediatric Surgery, Marciniak Hospital, Wroclaw, Poland; 8 Department of Paediatric Traumatology and Emergency Medicine, Medical University, Wroclaw, Poland; 9 Department of Diagnostic Imaging, St Jude Children’s Research Hospital, Memphis, TN USA

## Abstract

**Background:**

Our aim is to show whether the sentinel node procedure (SNP) is recommendable for pediatric patients with extremity rhabdomyosarcoma (RMS). Lymph node metastases are an important prognostic factor in pediatric patients with extremity RMS. Accurate nodal staging is necessary to treat the patient accordingly. An alternative to the current recommended lymph node sampling is the sentinel node procedure (SNP).

**Methods:**

A systematic review was performed summarizing all published cases of SNP in addition to 13 cases from our hospital and 8 cases from two other hospitals that have not been published before.

**Results:**

For all patients (*n* = 55), at least one SLN was identified, but the SNP technique used was not uniform. The SNP changed the nodal classification of eight patients (17.0%) and had a false-negative rate of 10.5%.

**Conclusions:**

The SNP is recommendable for pediatric patients with extremity RMS. It can change lymph node status and can be used to sample patients in a more targeted way than nodal sampling alone. Therefore, we recommend use of the SNP in addition to clinical and radiological nodal assessment for pediatric patients with extremity RMS.

Lymph node metastases are a concern in pediatric patients with extremity RMS, the most common childhood soft tissue sarcoma.^1^ The presence of regional lymph node metastases, which occur in 14–38% of extremity RMS patients,^2^–^5^ results in a significant drop in the 5-year overall survival rate from approximately 71 to 51%.^6^ Hence, accurate detection of involved lymph nodes is required to classify patients into the correct risk group with its associated prognosis and therapeutic approach.^2^,^7^

Unfortunately, clinical and radiological assessment of lymph node status is not always sufficient since regional lymph node biopsy changed lymph node status in 16–17% of extremity RMS patients, as shown by both the EpSSG-RMS2005 study and Intergroup Rhabdomyosarcoma Study Group IV.^5^,^8^ Therefore, nodal sampling is currently recommended as an integral part of staging extremity RMS. Nevertheless, the Surveillance, Epidemiology, and End Results (SEER) database showed that, in 74% of extremity RMS cases, nodal sampling was not performed according to protocol, reducing overall survival.^9^

An alternative to random nodal sampling is the sentinel node procedure (SNP). The targeted approach of the SNP provides more guidance to surgeons than random sampling. The SNP has been shown to be a reliable tool for nodal staging of melanoma and breast cancer patients.^10^,^11^ Currently, the SNP is not routinely used in extremity RMS patients because of the lack of evidence for its feasibility in nodal staging of these patients.

We performed a systematic review which summarizes all published cases of the SNP in pediatric extremity RMS patients and includes 13 cases from our hospital and 8 cases from two other hospitals that have not been previously published. We thereby aim to establish whether the SNP is recommendable. In addition, possible procedural concerns are discussed based on a recent case from our clinic.

## Methods

### Search Strategy

We performed an extended search in PubMed, Embase, CINAHL, and Web of Science, without restriction on publication year and with the latest update on 5 December 2019.^12^ The search strings were developed with the assistance of a medical librarian and are described in “Appendix”. The systematic search was finalized by hand searches and snowballing. Titles, abstracts, and full texts were screened by two authors (S.T., J.M.) using predefined inclusion and exclusion criteria. Disagreements were resolved by discussion. All original research articles that described the SNP in patients with extremity RMS between the age of 0 and 30 years were included. Articles written in English language were included. Because of the small number of studies expected to be identified, we included case reports and cohort studies. Patients with RMS of the thigh cranial from the trochanter complex or buttock were excluded because drainage is often to iliac and paraaortic lymph nodes that would not be accessible for the SNP.

### Data Extraction

The following data were extracted: type of study, patient characteristics (sex, age), tumor histology, tumor site [arm (proximal or distal), hand, leg (proximal or distal), foot], availability of preoperative imaging, nodal status according to radiology as performed in current practice (positive or negative), tracer agent (blue dye and/or radiocolloid), method of injection (peritumoral or intradermal), number of SLNs, location of SLNs, nodal status according to the SNP (positive or negative), presence of relapses, time to relapse, pattern of relapse (local, regional, or distal), survival status, and duration of follow-up. When articles did not report data important for this review, authors were contacted to provide this raw data.

### Procedure

The technique of the SNP consists of peritumoral or intradermal injection of a radiocolloid such as technetium-99m sulfur colloid and, if optical guidance is desirable, blue dye just before surgery. Lymphoscintigraphy is performed preoperatively. Lymph nodes that are radioactive and/or blue are resected, and presence of metastatic tumor cells is evaluated by a pathologist.^13^,^14^

### Definitions of Outcomes

A SLN is defined as the first blue and/or radioactive lymph node (or nodes) on a direct lymphatic drainage pathway from a primary tumor site. A positive SLN is defined as a harvested SLN that contains tumor cells as demonstrated by pathological evaluation according to current standards. We defined regional lymph nodes as those in the axilla or the groin, and in-transit nodes as those between the primary tumor and the regional node basin.

We defined a case as false negative if the patient developed a regional relapse while the SLN at that site was negative at diagnosis. This is according to the false-negativity definition used in the study of Wright et al.^15^ The false-negative rate is defined as the number of false negatives divided by the total number of false negatives and true positives.

Local failure is defined as relapse at the primary tumor site. Regional failure is defined as relapse in a regional draining lymph node, which includes in-transit metastases. Evidence of lymph node involvement beyond the regional lymph nodes is interpreted as distant metastasis.

### Statistics

The Kaplan–Meier methodology was employed to estimate overall survival (OS) for N0 and N1 patients^16^ (R version 3.5.2). Nodal staging was based on two different classifications systems: the SNP, and combined clinical and radiological assessment. For each classification system, survival curves for N0 and N1 patients were estimated. The log-rank method was used to assess the difference between survival curves within each classification system.

Cohen’s kappa was calculated to determine the agreement between the two nodal classification systems: SNP and radiology (SPSS version 25.0.0.2).

## Results

### Search and Screening

**Fig. 1:**
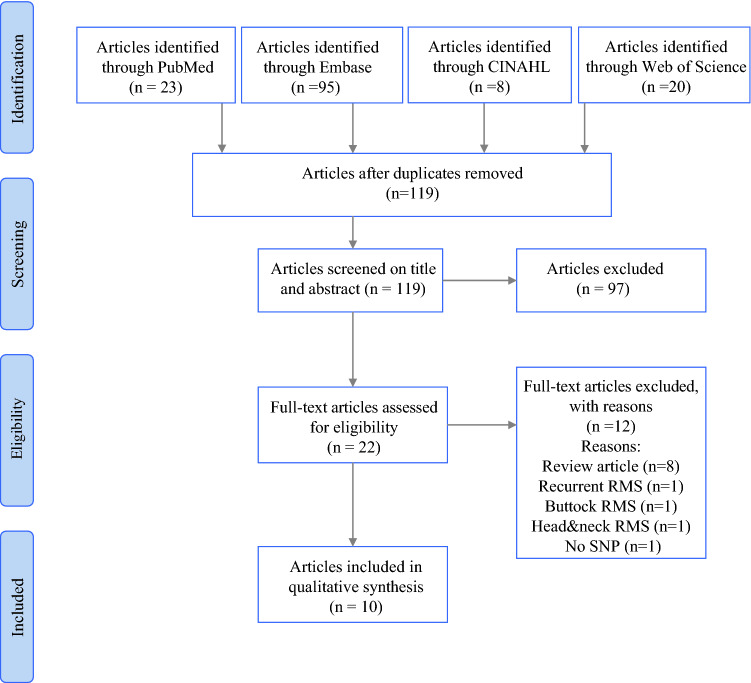
PRISMA flowchart depicting the flow of information through the different phases of this systematic review

A total of 146 articles were identified in PubMed (*n* = 23), Embase (*n* = 95), CINAHL (*n* = 8), and Web of Science (*n* = 20). After removal of duplicates, a total of 119 articles were screened on title and abstract. After full-text screening, a total of ten articles were included. The screening process is visualized in Fig. 1. There were four prospective cohort studies,^4^,^17^–^19^ five retrospective studies,^20^–^24^ and one case study.^25^ In the articles of De Corti and Dall’igna, some patients were mentioned in both studies.^17^,^26^ We included those patients once. In addition, we included extremity RMS patients who were treated at our hospital (*n* = 13), St. Jude’s Children’s Research Hospital (*n* = 5), and Marciniak Hospital (*n* = 3) who were not included in previous publications.

### Patient Description, Tumor Histology, and SNP Indication

In total, 55 patients with localized extremity RMS who underwent a SNP were described (Table 1). The median age of these patients was 8.0 years (interquartile range 3.0–14.8 years), and 54.9% were female. Alveolar RMS occurred in 40 patients (72.7%), 4 patients (7.3%) had embryonal RMS, 1 patient (1.8%) had pleomorphic RMS, and 1 patient (1.8%) had infantile spindle cell RMS, while for 9 patients (16.4%) histological subtype was not specified.

**Table 1:** Characteristics of 55 patients with localized extremity rhabdomyosarcoma who underwent a sentinel node procedure

References	Age (years)	Sex (M/F)	Histology	Tumor site	Lymph radiology	Lymph radiology (positive or negative)	Metastasis in SLN (yes or no)	Location of SLNs	Number of SLNs	Was SLN false negative?	Metastasis
Alcorn^20^	13	M	Alveolar	Lower leg	Yes	Negative	No	Inguinal	1	No	Local and distant
	2	F	Alveolar	Lower leg	Yes	Negative	No	Inguinal and popliteal	2	No	Local
	1	M	Alveolar	Lower arm	Yes	Positive	No	Axilla	1	No	None
	17	M	Alveolar	Lower leg	Yes	Negative	Yes	Inguinal	1	No	Distant
Andreou^19^	26	M	Pleiomorf	Leg	Yes	Negative	No	Inguinal	5	No	None
De Corti^18^	6	F	Alveolar	Lower arm	Yes	Negative	No	Axilla	2	No	None
	7	F	Alveolar	Lower arm	Yes	Negative	No	Axilla	1	No	None
	15	F	Alveolar	Arm	Yes	Negative	No	Axilla	1	No	None
	15	F	Alveolar	Hand	Yes	Positive	Yes	Axilla	5	No	Distant
	2	F	Embryonal	Leg	Yes	Negative	No	Inguinal	2	No	None
Dall’Igna^17^	7	F	Alveolar	Lower arm	Yes	Negative	No	Axilla	3	No	None
	13	F	Alveolar	Lower arm	Yes	Positive	No	Axilla	1	No	None
	16	F	Alveolar	Hand	No	X	Yes	Axilla	2	No	Regional and distant
	9	M	Alveolar	Leg	Yes	Positive	No	Inguinal	3	No	None
	9	M	Alveolar	Leg	Yes	Negative	No	Inguinal	2	No	None
Gow^21^	2	F	Alveolar	Arm	Yes	Negative	No	Axilla	6	No	None
	13	M	Alveolar	Leg	Yes	Negative	No	Inguinal	2	No	None
	2	M	Alveolar	Leg	Yes	Negative	Yes	Inguinal	3	No	None
Kayton^22^	7	F	Alveolar	Lower leg	Yes	Positive	No	Inguinal	X	No	Local
	12	M	X	X	No	X	No	X	X	No	None
	8	M	X	X	No	X	No	X	X	No	None
	4	M	X	X	No	X	No	X	X	No	Local
	17	M	X	X	No	X	No	X	X	Yes	Regional and distant
	16	F	X	X	No	X	Yes	X	X	No	Distant
McMulkin^25^	6	M	Alveolar	Hand	Yes	Negative	No	Elbow and axilla	13	No	None
Neville^23^	4	F	X	Lower arm	X	X	No	X	1	No	None
	2	F	X	Lower arm	X	X	Yes	X	2	No	None
Parida^24^	16	F	Alveolar	Hand	Yes	Negative	No	Axilla	3	No	None
	12	F	Alveolar	Lower arm	Yes	Negative	No	Elbow and axilla	5	No	None
	7	F	Alveolar	Upper leg	Yes	Positive	Yes	Inguinal and iliacal	2	No	None
	18	M	Embryonal	Hand	Yes	Negative	No	Axilla	2	No	None
	X	F	X	X	Yes	Negative	No	X	X	No	None
	X	F	X	X	Yes	Negative	No	X	X	No	None
Wagner^4^	7	F	Alveolar	Lower arm	Yes	Negative	No	Axilla	5	No	None
Godzin (2020) unpublished	14	F	Alveolar	Lower arm	Yes	Positive	Yes	Elbow and axilla	2	No	Local
	8	M	Alveolar	Lower arm	Yes	Negative	No	Axilla	2	No	None
	15	M	Alveolar	Lower leg	Yes	Positive	Yes	Inguinal and iliacal	2	No	None
Shulkin (2020) Unpublished	2	F	Spindle-cell	Arm	Yes	Negative	No	Axilla	10	No	None
	15	M	Alveolar	Hand	Yes	Positive	Yes	Elbow and axilla	4	No	None
	15	F	Alveolar	Hand	Yes	Positive	Yes	Axilla	1	No	None
	8	M	Alveolar	Hand	Yes	Positive	Yes	Elbow and axilla	2	No	None
	X	M	Alveolar	Hand	Yes	Positive	Yes	Axilla	3	No	None
Terwisscha (2020) Unpublished	18	F	Embryonal	Lower arm	Yes	Negative	No	Axilla	2	No	None
	1	M	Alveolar	Lower arm	Yes	Positive	Yes	Axilla	3	No	Regional and distant
	8	M	Alveolar	Upper leg	Yes	Negative	No	Inguinal	3	Yes	Regional and distant
	3	M	Alveolar	Lower leg	Yes	Positive	No	Inguinal	1	No	None
	2	F	Alveolar	Lower arm	Yes	Negative	No	Axilla	1	No	Distant
	3	F	Alveolar	Upper leg	Yes	Negative	No	Inguinal	1	No	None
	11	F	Alveolar	Upper leg	Yes	Negative	No	Inguinal	3	No	None
	3	F	Alveolar	Lower leg	Yes	Positive	Yes	Popliteal	1	No	None
	11	F	Alveolar	Lower arm	Yes	Negative	No	Axilla	2	No	None
	13	F	Alveolar	Lower leg	Yes	Positive	Yes	Inguinal and popliteal	3	No	None
	1	F	Alveolar	Upper leg	Yes	Positive	No	Inguinal	2	No	None
	5	F	Alveolar	Lower arm	Yes	Negative	No	Axilla	1	No	None
	2	M	Embryonal	Hand	Yes	Positive	Yes	Elbow and axilla	2	No	None

*SLN* sentinel lymph node

### Technique of SNP

The full SNP technique was described for 47 patients (85.5%) (Table 2). Radiocolloid tracer was used for 54 patients (98.2%). Blue dye was used for 38 patients (69.1%), not used for 11 patients (20.0%), and unknown for 6 patients (10.9%). Tracer injection was intradermal for 24 patients (43.6%), peritumoral for 29 patients (52.7%), and unknown for 2 patients (3.6%).

**Table 2:** The sentinel node procedure technique as described per patient

References	Age (years)	Radiocolloid (yes or no)	Blue dye (yes or no)	Injection site
Alcorn^20^	13	Yes	Yes	Intradermal
	2	Yes	Yes	Intradermal
	1	Yes	Yes	Intradermal
	17	Yes	Yes	Intradermal
Andreou^19^	26	Yes	Yes	Intradermal
De Corti^18^	6	Yes	Yes	Intradermal
	7	Yes	Yes	Intradermal
	15	Yes	Yes	Intradermal
	15	Yes	Yes	Peritumoral
	2	Yes	Yes	Intradermal
Dall’Igna^17^	7	Yes	Yes	Intradermal
	13	Yes	No	Intradermal
	16	Yes	No	Intradermal
	9	Yes	No	Intradermal
	9	Yes	No	Intradermal
Gow^21^	2	Yes	Yes	Intradermal
	13	Yes	Yes	Intradermal
	2	Yes	Yes	Intradermal
Kayton^22^	7	Yes	X	Intradermal
	12	Yes	X	Intradermal
	8	Yes	X	Intradermal
	4	Yes	X	Intradermal
	17	Yes	X	Intradermal
	16	Yes	X	Intradermal
McMulkin^25^	6	Yes	Yes	Intradermal
Neville^23^	4	Yes	Yes	X
	2	No	Yes	X
Parida^24^	16	Yes	Yes	Peritumoral
	12	Yes	Yes	Peritumoral
	7	Yes	Yes	Peritumoral
	18	Yes	Yes	Peritumoral
	X	Yes	Yes	Peritumoral
	X	Yes	Yes	Peritumoral
Wagner^4^	7	Yes	Yes	Peritumoral
Godzin (2020) unpublished	14	Yes	No	Peritumoral
	8	Yes	No	Peritumoral
	15	Yes	No	Peritumoral
Shulkin (2020) Unpublished	2	Yes	Yes	Peritumoral
	15	Yes	No	Peritumoral
	15	Yes	No	Peritumoral
	8	Yes	No	Peritumoral
	X	Yes	No	Peritumoral
Terwisscha (2020) Unpublished	18	Yes	Yes	Peritumoral
	1	Yes	Yes	Peritumoral
	8	Yes	Yes	Peritumoral
	3	Yes	Yes	Peritumoral
	2	Yes	Yes	Peritumoral
	3	Yes	Yes	Peritumoral
	11	Yes	Yes	Peritumoral
	3	Yes	Yes	Peritumoral
	11	Yes	Yes	Peritumoral
	13	Yes	Yes	Peritumoral
	1	Yes	Yes	Peritumoral
	5	Yes	Yes	Peritumoral
	2	Yes	Yes	Peritumoral

### SLN detection

**Fig. 2:**
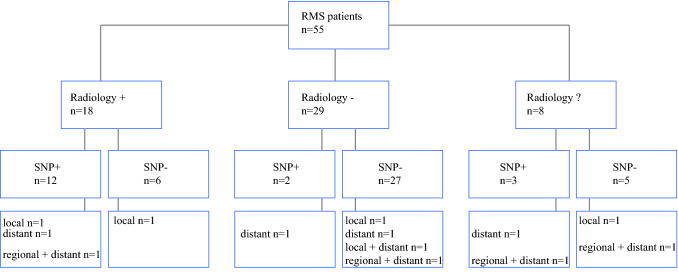
Flowchart presenting the number of RMS patients with a positive sentinel lymph node (SLN) according to radiology and/or the sentinel node procedure (SNP) including the number of relapses per group

In all patients, at least one SLN was detected. The median number of SLNs reported was 2 (interquartile range 1–3, range 1–13). In 83.7% of patients, the SLN location was reported. In patients with a distal extremity tumor, 28.1% had an in-transit SLN (Table 1). Figure 2 shows which patients were found to have positive nodal disease with either radiology and/or the SNP.

To perform an accurate SNP, it is important to understand the lymph drainage in extremity RMS patients. To this end, we discuss one of the patients treated at our hospital. This 13-year-old patient presented with alveolar RMS of the calf. Magnetic resonance imaging (MRI) showed suspected but not very enlarged popliteal nodes. We decided to sample the popliteal nodes but also to perform a SNP. Tc99 nanocolloid was injected at four sites around the tumor, and scintigraphy was performed, revealing deep drainage along the vessels to the popliteal nodes and from there to an inguinal node. Another more superficial drainage pathway was seen directly from the tumor site to a second inguinal node. Sampling of these SLNs showed metastases in the popliteal nodes (following the deep drainage) but not in the inguinal second-echelon node. Micrometastases were found in the inguinal SLN following the superficial pathway. This shows that, at tumor sites in the lower leg and forearm, it is important to consider both the deep and superficial drainage systems, which can only be identified separately using the SNP.

### Clinical Consequences

In 47 patients, both radiological and SNP nodal status were known. Two of these patients (4.3%) were upstaged from N0 to N1 by the SNP. These patients had no radiological evidence of regional metastatic disease. Six of these patients (12.8%) were downstaged from N1 to N0 by the SNP. These patients had radiological evidence of regional metastatic disease, but the SNP was shown to be negative. Unless otherwise reported, the SLN was considered to correspond to the radiologically suspected node. In total, the nodal classification changed for eight patients (17.0%).

The false-negative rate (FNR) was calculated by dividing the false-negative cases by the sum of the false-negative cases plus the true-positive cases. Four cases had regional relapse. Two of them had a SLN at that site that was negative. These were considered false-negative cases. Seventeen patients had a true-positive SLN. Therefore, the FNR of the SNP was 10.5% (2/19).

Patients with a positive SLN had any relapse in 35.3% (6/17), local relapse in 5.9% (1/17), distant metastasis in 17.6% (3/17), and regional + distant metastasis in 11.8% (2/17) (Fig. 2). Patients with a negative SLN had any relapse in 18.4% (7/38), local relapse in 7.9% (3/38), distant metastasis in 2.6% (1/38), local + distant metastasis in 2.6% (1/38), and regional + distant metastasis in 5.3% (2/38).

### Survival and Agreement Between Classification Systems

**Fig. 3:**
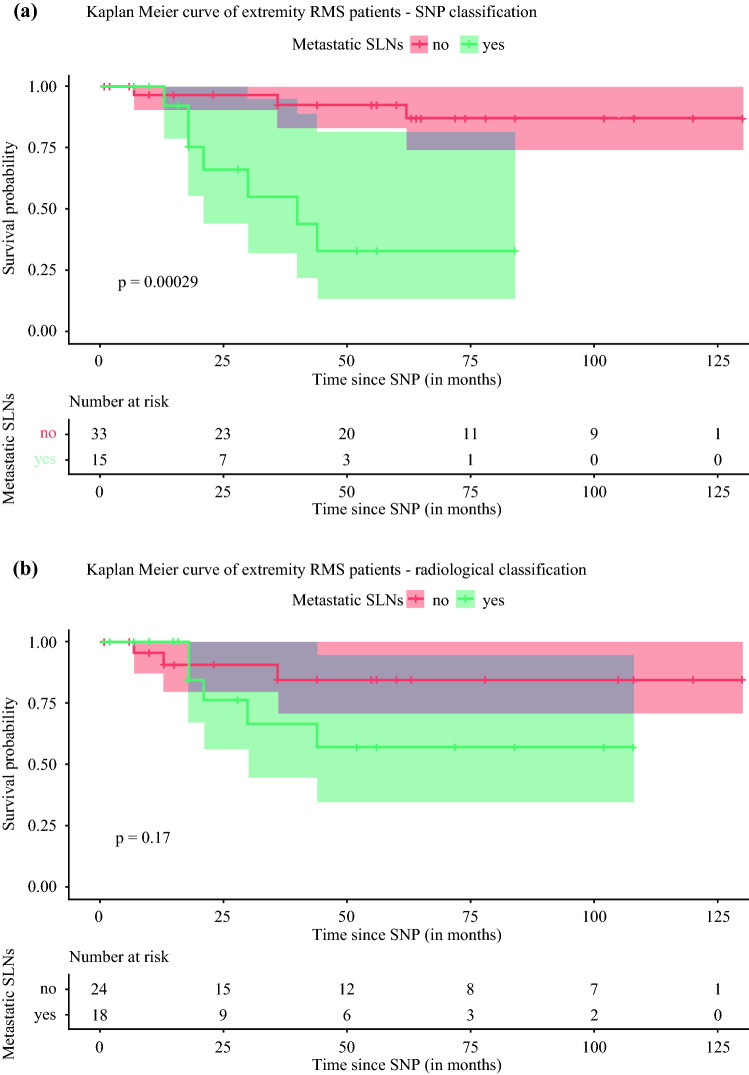
Kaplan–Meier curve of extremity RMS patients, with nodal stage based on the sentinel node procedure (SNP) or radiology. *SLN* sentinel lymph node, *LN* lymph node

When nodal stage was based on the SNP, the estimated overall survival (OS) at 2 years was 96.7% [95% confidence interval (CI) 90.1–100%] and 66.1% (95% CI 43.7–99.9%) for N0 and N1 patients, respectively (Fig. 3). OS at 5 years was 92.5% (95% CI 82.9–100%) and 33.0% (95% CI 13.4–81.3%) for N0 and N1 patients, respectively. Differences between the two survival curves were assessed by log-rank test. Classification based on SNP showed a statistically significant difference in survival (*p* < 0.01). When nodal stage was based on radiology, the 2-year OS was 90.7% (95% CI 79.2–100%) for N0 patients and 76.2% (95% CI 55.8–100%) for N1 patients. The 5-year OS was 84.6% (95% CI 69.9–100%) for N0 patients and 57.1% (95% CI 34.4–94.8%) for N1 patients. No significant difference in survival was found (*p* = 0.17). Cohen’s kappa was 0.62, suggesting moderate agreement between the SNP and radiological nodal classification systems.

## Discussion

This is the first time that SNP data concerning all reported cases plus 21 unpublished cases, rendering a total of 55 patients, have been summarized. These data show that the SNP is recommendable in pediatric patients with extremity RMS.

The SNP changed the nodal classification of 17.0% of the patients (4.3% were upstaged from N0 to N1, and 12.5% were downstaged from N1 to N0). This is in accordance with the change in nodal stage as a consequence of nodal sampling in the EpSSG-RMS2005 study, although the ratio between up- and downstaging was different.^5^ Due to the changed nodal stage, these patients will be treated differently. This demonstrates the potential impact of the SNP on the accuracy of nodal classification in pediatric patients with extremity RMS.

Our cohort revealed an acceptable FNR of 10.5% for the SNP. This is considered acceptable, since it is in the FNR range for the SNP of breast cancer and melanoma patients (8.8–16.7%^10^,^27^ and 5.2–10.3%,^11^ respectively), which is a worldwide accepted treatment modality. The FNR values of melanoma and breast cancer SNPs were based on studies performing both the SNP and complete lymphadenectomy in the same patients. It is not ethically justified to repeat such a study design for this pediatric SNP indication as current therapy for lymph node metastases is not lymph node dissection, but radiotherapy. Therefore, we based the FNR on data regarding relapses in regional lymph nodes that was supplied by the authors or their colleagues.

The presence of false-negative cases may be partly explained by the fact that the SNP is only able to identify synchronous regional metastasis, not metachronous metastasis. Furthermore, the complex lymph drainage of extremity RMS could lead to false-negative cases. The lymph drainage of distal extremity RMS can occur via both the deep and superficial lymph drainage system, due to its location and mesenchymal origin. Therefore, these tumors can be associated with different metastatic patterns, as demonstrated by our case report. This patient had a SLN in both the deep (popliteal SLNs) and superficial lymph drainage system (inguinal SLN). Due to the direct lymph drainage via two different pathways, both SLNs are considered primary SLNs and not second-echelon nodes. In this case, the SLNs in both basins were shown to contain metastatic cells and led to preoperative radiotherapy of both nodal basins. Radiotherapy would have been inadequate if one of these positive SLN basins had not been identified. Furthermore, due to the potential dual lymph drainage (deep and superficial) of distal extremity RMS, we do not recommend the use of clinically or radiologically suspected nodes as a contraindication to perform a SNP for this indication. In addition, any nonsentinel node that is clinically or radiologically suspected to contain metastasis should always be biopsied, as pathologically enlarged nodes can alter lymphatic drainage and lead to a different SLN. Therefore, a pathologically enlarged lymph node can be bypassed with the SNP, and imaging remains important.

Finally, our results show that the nodal stage based on the SNP classification is of prognostic value for the estimated OS, while that is not the case for the radiological classification. When patients were staged into N0 or N1 according to the SNP, the estimated overall survival (OS) curves differed significantly. The 5-year OS was 92.5% (95% CI 82.9–100%) for N0 patients but only 33.0% (95% CI 13.4–81.3%) for N1 patients. On the contrary, when the same patients were staged into N0 or N1 based on the radiological assessment, there was no significant difference in estimated OS. This shows the importance of nodal sampling in extremity RMS patients in addition to radiological imaging.

Currently, there is no consensus about the optimal method to visualize both the deep and superficial lymph drainage systems. The injection site, either intradermal or peritumoral, may matter. The current numbers concerning the injection site are too small to answer this question. However, there are two reasons that peritumoral injections seem to be more logical. First, lymph drainage depends on the localization of the tumor, and localization of RMS is very diverse, from (sub)cutaneous to bone marrow. Secondly, RMS is of mesenchymal origin, while the dermis is of ectodermal origin.^28^

Since this is a retrospective study, some data were unknown. We could not distinguish between different imaging modalities because they are based on the current radiology practices of the various institutes. Also, in case of a radiologically suspected lymph node, we assumed that the SLN corresponded to that lymph node, unless otherwise reported. Furthermore, there is a risk of selection bias (i.e., the patients in whom a SNP is performed may not be representative of the whole population of extremity RMS patients).

A second limitation of this study is that surgical SNP expertise has increased over the years, possibly influencing the procedural success. However, this will probably not significantly influence our main results, since there was at least one SLN resected in every SNP. Finally, we were not able to compare SNP with the current standard, random sampling; therefore, although theoretically superior, we cannot make firm conclusions on the comparison of SNP with random sampling.

To conclude, for nodal assessment of extremity RMS patients, it is clear that the pathological assessment of a lymph node is preferable to clinical and radiological evaluation alone. There are two methods that allow pathological assessment: the SNP and random nodal sampling. With the currently available data, we are unable to compare these two methods directly. This would require a prospective randomized study. Nevertheless, it is questionable whether such a study should be conducted, given its feasibility (number of patients that need to be randomized) and additive value. One can expect from such a study that the SNP (in addition to sampling of suspected lymph nodes) is either noninferior or superior to random nodal sampling, since it is targeted. In addition, it can be expected that surgeons’ compliance to the protocol will improve with the SNP, as this is a more rational approach with less risk of complications from unnecessary lymph node removal. Therefore, we recommend use of the SNP in addition to clinical and radiological assessment for nodal staging of pediatric patients with extremity RMS, even in case of clinically or radiologically suspected nodes. Given the diverse anatomical locations and the mesenchymal origin of RMS, the use of peritumoral injections seems to be most logical but has not been proven superior to intradermal injections so far.

## Appendix: Search Strategy

### PubMed

(sentinel node*[tw] OR sentinel lymph*[tw] OR Lymphatic map*[tw] OR sentinel ln*[tw] OR sentinel limph*[tw] OR nonsentinel lymph*[tw] OR nonsentinel node*[tw])

### AND

(child[tw] OR children[tw] OR teen*[tw] OR toddler*[tw] OR preschool*[tw] OR infant*[tw] OR childhood[tw] OR juvenile*[tw] OR ((adolescen*[tw]) NOT adult[mesh]) OR pediatric*[tw] OR paediatric*[tw])

### AND

(Sarcom*[tw] OR Rhabdomyosarcom*[tw])

### Embase

((sentin* OR nonsentin* OR map*) NEAR/3 (node* OR ln* OR lymph* OR limph*)):de,ab,ti AND ((child* OR teen* OR toddler* OR preschool* OR infant* OR juvenile* OR pediatric*):de,ab,ti OR ((adolescen*):de,ab,ti NOT adult/exp)) AND (Sarcom* OR Rhabdomyosarcom*):de,ab,ti

### WoS

((sentin* OR nonsentin* OR map*) NEAR/3 (node* OR LN OR LNs OR lymphnode* OR limph*)) AND ((child* OR teen* OR toddler* OR preschool* OR infant* OR juvenile* OR pediatric*) OR ((adolescen*) NOT adult)) AND (Sarcom* OR Rhabdomyosarcom*)

### CINAHL

((sentin* OR nonsentin* OR map*) N3 (node* OR ln* OR lymphnode* OR limph*)) AND ((child* OR teen* OR toddler* OR preschool* OR infant* OR juvenile* OR pediatric*) OR ((adolescen*) NOT adult)) AND (Sarcom* OR Rhabdomyosarcom*)
